# Prevalence of pediatric lower urinary tract symptoms in a US population seeking medical care, 2003- 2014

**DOI:** 10.21203/rs.3.rs-2883579/v1

**Published:** 2023-06-05

**Authors:** Kathleen M. Kan, Gunjan Agrawal, Raphael Brosula, Pranaya Venkatapuram, Abby L. Chen, Chiyuan A. Zhang

**Affiliations:** Stanford University School of Medicine; Flushing Hospital Medical Center; Stanford University School of Medicine; Stanford University School of Medicine; Stanford University School of Medicine; Stanford University School of Medicine

**Keywords:** Lower urinary tract symptoms, population health research, pediatrics

## Abstract

**Background:**

We conducted this study to estimate the prevalence of pediatric lower urinary tract symptoms (pLUTS) in a US privately-insured pediatric population who are 18 years of age or older by age, sex, race/ethnicity from 2003–2014. This has not been previously described in the literature.

**Methods:**

We retrospectively reviewed Optum’s de-identifed Clinformatics^®^ Data Mart Database database between 2003–2014. A pLUTS patient was defined by the presence of ≥ 1 pLUTS-related ICD-9 diagnosis code between the age of 6–20 years. Neurogenic bladder, renal transplant and structural urologic disease diagnoses were excluded. Prevalence by year was calculated as a proportion of pLUTS patients among the total population at risk. Variables reviewed included age, sex, race, geographic region, household factors and clinical comorbidities including attention-deficit/hyperactivity disorder (ADHD), constipation, and sleep apnea. Point of service (POS) was calculated as a proportion of pLUTS-related claims associated with a POS compared to the total claims at all POS in the time period.

**Results:**

We identified 282,427 unique patients with ≥ 1 claim for pLUTS between the ages of 6–20 years from 2003–2014. Average prevalence during this period was 0.92%, increasing from 0.63% in 2003 to 1.13% in 2014. Mean age was 12.15 years. More patients were female (59.80%), white (65.97%), between 6–10 years old (52.18%) and resided in the Southern US (44.97%). Within a single household, 81.71% reported ≤ 2 children, and 65.53% reported ≥ 3 adults. 16.88% had a diagnosis of ADHD, 19.49% had a diagnosis of constipation and 3.04% had a diagnosis of sleep apnea. 75% of pLUTS-related claims were recorded in an outpatient setting.

**Conclusions:**

Families consistently seek medical care in the outpatient setting for pLUTS. The demographic and clinical characteristics of our cohort reflect prior literature. Future studies can help define temporal relationships between household factors and onset of disease as well as characterize pLUTS-related healthcare resource utilization. Additional work is required in publicly-insured populations.

## Introduction

Pediatric lower urinary tract symptoms (pLUTS) has remained a common childhood disease in the United States. pLUTS includes a range of presentations in children older than 5 years who have completed potty training, such as daytime and nighttime incontinence, urgency, frequency, and dysuria.^1^ Factors associated with disease presentation include gender, family history of incontinence, constipation and behavioral disorders such as Attention-deficit/hyperactivity disorder (ADHD).^2-4^ Sequelae of disease includes decrease in quality of life and self-esteem as well as recurrent urinary tract infection.^5,6^

An estimated 36–128 million dollars/year are spent on pediatric incontinence using Healthcare Cost and Utilization Project (HCUP) data.^7^ Data estimating prevalence of pLUTS has largely relied on cross-sectional survey methods. Three large studies found daytime urinary incontinence (DUI) in 10% of children sampled, though differences in study techniques and questionnaires limits direct comparison of findings.^8-10^ pLUTS symptoms within these DUI populations were higher with participants reporting urgency, frequency and voiding postponement behaviors. ^8-10^

The number of children with pLUTS seeking medical care and how they use medical resources has not been well-defined. Existing HCUP data estimates a rate of approximately 1,000 per 100,000 children ages 3–10 years requiring outpatient care for a diagnoses of pediatric incontinence per year.^7^ No data exists on additional drivers of healthcare utilization such as household factors or clinical co-morbidities. In order to understand disease burden, longitudinal risk factors, and design effective population-level treatment strategies, it is necessary to further characterize this patient population.

Optum’s de-identifed Clinformatics^®^ Data Mart Database (hereafter, referred to as CDM database) is a US database with pharmaceutical and health claims of commercially insured individuals across all 50 states. Both the pediatric population and outpatient setting are represented. Results may be limited by differences in population characteristics in a commercially insured versus uninsured group, however this dataset still represents a unique opportunity to explore a pLUTS cohort using claims data that can be used for future healthcare resource utilization studies. We aim to define the prevalence of individuals with pLUTS who are seeking medical care from 2004–2014 within the CDM database, and characterize point of service, demographic factors and clinical comorbidities of constipation and ADHD within this cohort.

## Methods

### Data source:

After obtaining institutional review board approval, we analyzed data derived from the CDM database between the years 2003–2014. Clinformatics^®^ Data Mart Database is a de-identified database is derived from a large adjudicated claims data warehouse. The database includes deidentified administrative health claims for members of large commercial and Medicare Advantage health plans, approximately 15–20 million annual covered lives. Enrollment, demographic and healthcare claims data related to outpatient services and emergency room visits are available. Diagnoses were coded using the International Classification of Diseases, ninth revision (ICD-9), procedures using Current Procedural Terminology (CPT).

### Study population:

We conducted a retrospective population-based cohort study. Our cohort included all patients ages 6–20 years old at any time between 2003–2014 with an ICD-9 code for pLUTS (Appendix 1) similar to prior HCUP data.^7^ A patient was considered to have pLUTS if they had ≥ 1 diagnosis associated with any point of service claim (i.e. POS claim associated with an inpatient, outpatient, or emergency room visit). We excluded patients with an ICD-9 diagnosis or CPT surgical code related to neurogenic bladder, renal transplant, and structural urologic disease that increases risk for pLUTS - hypospadias, vesicoureteral reflux, posterior urethral valves, urethral stricture disease and ureterocele. The total population at risk per year between the ages 6–20 years old in the database was obtained from CDM database member data files version 7.0. To analyze demographic and clinical characteristics, we included all patients 6–20 years old at any time between 2003–2014 with an ICD-9 code for pLUTS.

Daytime and nighttime incontinence were defined by ICD-9 codes specific to incontinence. The remaining non-incontinence codes were grouped together as “other pLUTS.” Patient demographics, including age, sex, race, and clinical comorbidities, were collected. The primary outcome was prevalence of pLUTS per one year period between 2003–2014. Secondary outcomes were characterization of point of service, demographic variables, and clinical characteristics within the pLUTS population over the total time period. Demographic variables included age, race, geographic region, household income level of primary insurance provider, and number of household members. Clinical comorbidities included constipation and ADHD as defined by ICD-9 codes at any point in time. (Appendix 1)

### Statistical analysis:

Prevalence of pLUTS by year was calculated as a proportion of pLUTS patients among the total population at risk within a 6–20 year age group per each one-year period. Each pLUTS patient was counted once per year and could be counted the following year until they reached 20 years of age or left the database. Descriptive analysis of demographic characteristics and clinical comorbidities within the total pLUTS population were calculated as percentages over the total time period. All analysis was conducted using Excel, and descriptive summaries were obtained within Redivis.

## Results

**Figure 1:**
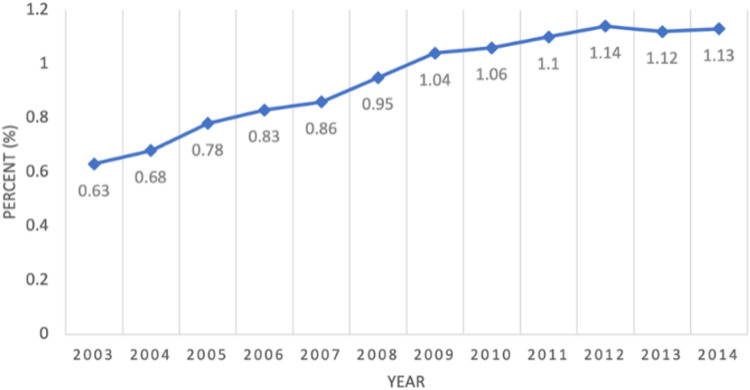
Prevalence by year of pLUTS from 2003-2014

We identified the total population at risk during this time period to be 9,263,933. After applying exclusion criteria, our total pLUTS cohort was 282,427. This data demonstrates a 0.92% average prevalence of pLUTS between 2003-2014. Yearly prevalence increased from 0.63% in 2003 to 1.13% in 2014 (Figure 1). Among patients who visited a medical facility for a pLUTS related diagnosis, office visits comprise the majority of total clinical encounters (59.70%), followed by outpatient hospital visits (15.27%), inpatient hospitalization (3.50%), and emergency room (ER) visits (2.16%) (Table 1).

Mean age of patients was 12.15 years with the proportion of patients in the 6-10 year old category (147,362, 52.18%). The majority of patients were female (59.80%), white (65.97%), and from the South (44.97%). Most families reported a household income >40k (45.47%) however we noted a 74% fill rate in this data. Among all households, 81.71% reported ≤2 children and 65.53% reported ≥3 adults. Daytime (28.33%) and nighttime (22.23%) incontinence diagnoses made up 50.56% of ICD-9 codes. Among all patients with pLUTS, 34.06% had a relevant clinical comorbidity. Of these diagnoses, constipation was the most common (19.49%), followed by ADHD (16.88%) (Table 1).

## Discussion

Average prevalence of pLUTS necessitating medical care in the CDM Database was found to be 0.92%, increasing from 0.63% in 2003 to 1.13% in 2014. We demonstrate the degree to which pLUTS burden is largely centered around outpatient care services (77.13%) compared to inpatient or emergency room visits. Most patients were younger, female and resided in the Southern US. ADHD and constipation rates mirror existing literature.^22^

Prevalence of pLUTS in the global community has been measured via cross-sectional survey methods. Chung et al. found “dysfunctional voiding” symptoms in 46.4% of Korean children ages 5-13.^9^ Type of presentations varied – 16.8% demonstrated urge incontinence, 16.6% urgency alone, 11.2% DUI, and 5.6% NE. ^9^ Lower urinary tract dysfunction (LUTD) was found in 9.3% of Turkish schoolchildren ages 6–15 years old and ^10^21.8% of Brazilian children. ^11^ Differences in prevalence can be attributed to differences in study population, age groups, and type of survey administered (validated vs non-validated) along respondent bias. Our use of a claims database relies on clinical assessment with rates less likely to be affected by factors such as likeliness to self-report, survey response rates, and recall bias.

An overall prevalence of 0.92% in the CDM database represents the percentage of privately insured pediatric patients seeking treatment for pLUTS. This prevalence is similar to rates seen in HCUP data of approximately 1,000 out of every 100,000 families seeking care for pediatric incontinence in age groups 3–10 years old within a year. ^7^ As expected, this number is lower than overall community prevalence estimates, as only 10–16% of families may seek medical care.^12^ While severity and frequency may be related to this decision, in a sub-group of children who experienced wetting daily, 40% had not received medical care. ^12 13^ Additional drivers such as lack of access to care, lack of knowledge regarding treatment options, or social norms that pLUTS is a condition to be outgrown, may play a role in treatment-seeking behaviors. ^13^

Of the total claims associated with a pLUTS diagnosis, 77.13% were performed in an outpatient setting. This is in contrast to the low numbers of patients requiring inpatient care (3.5%) for incontinence diagnoses, which reflect previously reported data for this point of service. ^7^ Our study found higher proportion of females (59%) and patients ages 6–10 years old (52.18%). While some studies have found a higher prevalence of LUTD and DUI in girls^13,14^, this has not been consistently demonstrated in other large studies. ^15^

Data from longitudinal studies of children with DUI found the highest prevalence of disease in early childhood with a spontaneous remission rate of 15.4% per year.^16^ Yuksel et al. found that the rates of LUTD stabilized around the ages of 10 in girl and 11 in boys, potentially representing pubertal changes.^10^ While the prevalence of disease in the community may drop in older children, we found that the next largest group of patients with pLUTS seeking medical care was 16 years or older (27%). This may reflect increased duration and/or severity of disease that prompts medical care or increasing independence in social activities that increases degree of bother.

Treatment of pLUTS consists of behavioral and lifestyle modifications to improve voiding, stooling and hydration habits. Effective application and adherence to these changes may be influenced by social determinants of health including availability of healthy foods, housing factors, and access to healthcare and education. Low levels of parental education, double-income families and household factors such as increased number of siblings or family members, increased number of people sleeping in the child’s room, were more likely to have pLUTS, however direct associations between low-income households, presence, or absence of insurance have not been observed.^9,10, 17,18^ Housing factors such as number of people per room or number of siblings may play a role in NE, potentially reflecting crowding at home that limits bathroom access. Families with fewer children and > 3 adults in the family had higher proportion of pLUTS diagnoses. This could represent lack of knowledge of pLUTS with fewer children, and household crowding factors, though this population generally fell above the federal poverty line.

Prevalence of constipation in the general population varies from 0.7–29.6%.^19^ It is higher within the pLUTS population, 34% of children with constipation experience NE and 29% experience DUI^20^. The bladder and rectum are situated next to each other in the pelvis, sharing similar innervation for urethral and anal sphincter control and higher pelvic floor tone leading to symptoms.^21^ Children who have a large stool burden may develop a distended rectum that stimulates detrusor muscle contractions, experienced as pain, urgency,incontinence,^21^ and fear of painful elimination. We found that 19.49% of children in our pLUTS population had a diagnosis of constipation and a higher proportion of children with pLUTS were found in Southern US regions, where higher rates of constipation are observed^22^. This may reflect factors that link both presentations together, such as diet or water consumption versus the above internal factors.

Behavioral problems, such as ADHD, are also comorbid with pLUTS presentations. Following a systematic review, an overall pooled estimate of ADHD prevalence in the community is 7.20%.^23^ A study of > 8000 children found a nearly double rate of externalizing problems related to attention and activity problems, oppositional behavior and conduct disorders in children with DUI versus those without DUI.^24^ In our cohort of children with pLUTS, 16.88% had a diagnosis of ADHD. This may be related to the secondary effects of wetting on the development of behavioral symptoms or psychological problems.^24^

The rates of comorbid constipation and ADHD are important to note due to the potential for these patients to experience lower compliance and less successful outcomes with treatment outcomes. Specific outreach and education programs may be required to address additional needs within these children.

Our description of patients seeking medical care for their symptoms has limitations in its application to a national population. Claims databases are subject to inaccurate and/or missing data.^25^ Our choice of pLUTS ICD codes is based on prior literature, however, sensitivity and specificity in the accuracy of coding has not been investigated for these diagnoses. In order to obtain a picture of healthy children seeking medical care for these diagnoses we excluded clinical conditions such as transplant and neurogenic bladder, however, the role of additional conditions such as developmental delay and presence of absence of urinary tract infection were not investigated.

The CDM database is a national sample of patients who are privately insured. It therefore represents a subset of a much larger population, specifically families who may be overall healthier and able to seek medical care. In this regard, our research question benefits from this cohort since we assume this is closer to the maximum number of patients who would seek care as opposed to an underestimate due to poor access to education or healthcare resources.

## Conclusion

In conclusion, a rate of approximately 1% annual prevalence of pLUTS in the CDM database points to a large and consistent burden to the healthcare system that would benefit from further studies. An improved understanding of clinical and demographic risk factors for pLUTS using longitudinal study methods in insured and those with limited access to insurance populations will help to inform effective treatment and prevention strategies. Further investigation into healthcare resource utilization using claims data will allow these programs to target areas of improvement to reduce healthcare spending and prompt investment into preventative programs.

## Figures and Tables

**Table 1:** Descriptive statistics of demographics, socioeconomic variables, comorbidities, and treatment among total number of unique pLUTS patients

Variables	N	Percentage
Total number of unique pLUTs patients	282,427	
Age		
6-10	147,362	52.18%
11-15	58,257	20.63%
16-20	76,808	27.20%
Sex		
Male	113,493	40.18%
Female	168,882	59.80%
Unknown	52	0.02%
Region		
Northeast	28,769	10.19%
Midwest	96,774	34.27%
South	127,002	44.97%
West	48,668	17.23%
Unknown	1,043	0.37%
Race		
White	186,324	65.97%
Hispanic	20,290	7.18%
Black	21,327	7.55%
Asian	6,926	2.45%
Unknown	47,560	16.84%
Household income		
<$40k	18,013	6.38%
>$40k	128,429	45.47%
Unknown	135,985	48.15%
Fed poverty level		
Below (Below 400% FPL)	1,387	0.49%
Above (Above 400% FPL)	145,055	51.36%
Unknown	135,985	48.15%
Number of children in household		
≤2	230,776	81.71%
>2	11,932	4.22%
Unknown	39,719	14.06%
Number of adults in household		
≤2	57,620	20.40%
>2	185,088	65.53%
Unknown	39,719	14.06%
Comorbid conditions		
ADHD	47,671	16.88%
Constipation	55,052	19.49%
Type of pLUTS		
Daytime Incontinence	80,000	28.33%
Nighttime Incontinence	62,771	22.23%
Other pLUTS	185,059	49.63%
Point of service		
Total	27,327,388	100.00%
Office	16,315,921	59.70%
Outpatient Hospital	4,174,574	15.27%
Inpatient Hospital	956,959	3.50%
Emergency Room	591,096	2.16%
Unknown	5,465,477	20.00%

## Abbreviations

pLUTS: pediatric lower urinary tract symptoms
ADHD: Attention deficit hyperactivity disorder
POS: point of service
ICD: International Classification of Diseases
CPT: Current Procedural Terminology
HCUP: healthcare utilization project
DUI: daytime urinary incontinence
CDM database: Clinformatics^®^ Data Mart
ER: emergency room
LUTD: lower urinary tract dysfunction
